# An Abscess in the Breast, Occasioned by a Fractured Rib, Attended
with Symptoms Supposed to Have Had a Cancerous Tendency, Successfully Treated;
and a Steatoma, Possessing the Greatest Part of the Neck on the Right
Side, Removed

**Published:** 1803-11-01

**Authors:** John Moodie

**Affiliations:** Senior Physician to the Bath City Dispensary


### *An* ABSCESS IN THE BREAST, *occasioned by a fractured Rib, attended with Symptoms supposed to have had a cancerous Tendency, successfully treated; and a* STEATOMA, *possessing the greatest Part of the Neck on the right Side, removed. By* JOHN MOODIE, M. D. &c. *Senior Physician to the Bath City Dispensary.*

A Lady of a delicate constitution, aged twenty-eight, had been some years subject to a cough, which returned with more or less violence every winter. She had also frequent attacks of sick head-ach, and a spasmodic affection, attended with fever, thirst, anxiety, and costiveness, with most of the symptoms which accompany the *asthma spasmodica* distinctly marked, and sometimes a diarrhœa.

In the course of an extensive practice, several cases of this kind have occurred, in the cure of which I have generally rally experienced the most beneficial effects from cold bathing. Having ordered the most powerful medicines, usually prescribed in similar complaints, to this patient, without the desired effect, I therefore advised the use of the cold bath in the summer and autumn months, which, together with a healthy residence in the country, entirely removed her disorder.

In treating of this subject, the account of Dr. Smollett's case, related by himself in the following lines, may not here be unworthy of public notice: "In consequence of a cold caught in France, I was seized with a violent cough, attended with fever and stitches in my breast, which tormented me all night long without ceasing; at the same time I had a great discharge by expectoration, and such a dejection of spirits as I never felt before. In this situation I took a step which may appear to have been dangerous: I knew there was no imposthume in my lungs, and I supposed the stitches were spasmodical; I was sensible that all my complaints were originally derived from relaxation; I therefore hired a chaise, and, going to the beach, about a league from the town, plunged into the sea without hesitation. By this desperate remedy I got a fresh cold in my head, but my fever and stitches vanished the very first day; and, by a daily repetition of the bath, I have diminished my cough, strengthened my body, and recovered my spirits."*

June, 1793, the lady aforementioned accidentally leaning over the ledge of a window, to remove a bird cage which had been placed on the outside, received a hurt about two inches below the right breast, which at the time was attended with considerable pain, and a sense of internal uneasiness. Upon examination soon after the accident, there appeared a small tumour and discoloration of the skin; the part was painful to the touch, but respiration was free; she had no sickness at stomach, or any other unfavourable symptoms. By the application of the *linimentum saponis cum opio*, in a short time the tumour diminished, and the part resumed its natural appearance.

Early in August she went to Weymouth for the benefit of sea bathing, but soon after her arrival at that place she was frequently seized with sickness at stomach, attended with violent retchings. Bleeding, sedatives, and a due attention to regimen were directed, but afforded no re-

* Smollett's Travels.

lief lief. Having remained about a month at Weymouth, and being unable to bathe, she returned to the neighbourhood of Bath; but, notwithstanding the use of such medicines as were deemed proper by the most eminent physicians of that place, the sickness at stomach and vomiting still continued. Upon examination of the side where she received the hurt, she now informed me that she felt a pricking pain, at times extremely acute, just below the right breast, where there appeared a small enlargement, but without any discolouration as formerly observed; I therefore had no doubt that one of the superior ribs had been fractured, or detached from its cartilaginous adhesion to the *sternum*, especially as she was sensible of the motion of the bones, which now and then occasioned great pain, accompanied with spasm, especially when she attempted to raise herself in bed. Leeches were repeatedly applied to the tumour, and rest in a horizontal position was enjoined. And when the inflammation was reduced, *emplast. thuris* with a moderately tight bandage was applied to the part; the body was kept gently laxative, and the following mixture directed:

℞. Mist. camp. ℥vß. spt. æther. vitriol. comp. ʒiß. tinct. opii. gutt. xxx. syr. tolutan. ʒiij. m. sumat. cochl. ij. ampl. subinde urgente spasmo.

About six weeks after the sickness at stomach and spasms were entirely removed, but there still remained a small enlargement of the breast, extending to the part where she received the hurt. However, nearly two years elapsed before there was sensible increase of the tumour; meanwhile she used the cold bath in the summer and autumn months, and generally enjoyed good health.

Toward the end of 1795, she at times complained of a pricking pain where she had received the hurt; the skin was somewhat discoloured, the breast considerably enlarged and uneven on its surface, attended with irregular pains shooting into the axilla and adjacent parts. A gentleman of the faculty recommended a discutient plaster to be applied to the tumour; after which it rapidly increased in size, with acute pain in the breast, and evident symptoms of the suppurative process going on. The lady having used considerable pressure upon the part by means of tight bandage, the fluid made its way into the breast, where it appeared to be contained in different cysts, which were moveable and circumscribed, and the glands in the axilla became greatly enlarged.

January, 1796, two eminent surgeons were consulted, and and were of opinion that the disorder had a cancerous tendency; however, they thought it might be advisable to try the effects of some powerful discutient previous to the operation. Accordingly the following cataplasm was directed warm to the part, and renewed twice a day:

℞. Sal. ammon. crud. ʒij. acet. vin. alb. ʒiv. aq. pur. ℥iv. farin. lini. q. s. m. et fiat cataplasm.

The above having been continued about a fortnight without any alteration, a blister was applied to the most depending part of the tumour, and kept open near three weeks with *ceratum sabinæ;* the discharge was very considerable, but the pain and irritation reduced the strength of the patient, and occasioned such violent spasms, that it was thought prudent to discontinue the use of the cerate.

At a second consultation, the gentlemen abovementioned were unanimously of opinion, that there could be no doubt of the tumour being a real cancerous affection of the breast, and contended that it had a connection with a steatomatous enlargement of her neck, which they imagined proceeded from a scrophulous habit, the tumour being at this time much inflamed and enlarged.

The gentlemen were therefore of opinion, that immediate recourse should be had to the operation, in order to remove such part of the breast as should be found cancerous. Being myself of a different opinion, I requested the operation might be deferred about a week, especially as it appeared to me that Nature was then making an effort which I had no doubt would soon relieve the patient. Emollient cataplasms were now applied twice a day to the breast, and the bark, in substance, was ordered to be taken freely.

On the 29th of March, when raising herself in bed, an opening was observed in the abscess large enough to admit a common probe, from whence issued a small quantity of lymphatic fluid; but as the opening was not sufficient to allow the tumour to empty itself freely, I made a puncture with a lancet in a longitudinal direction, about three quarters of an inch in extent, at the thinnest and most depending part of the abscess, which instantly discharged about a pint and a half of well digested pus tinged with blood. A pledget of digestive was applied to the orifice, and the poultice continued some days. The lady being much weakened by the discharge, a nourishing diet with port wine was recommended, and a drachm of Peruvian bark in powder four times a day, with an opiate at bed time.

Soon after, several small exfoliations from the rib, extremely irregular on their surfaces, and of a carious appearance, passed through the orifices, which continued open, with a considerable discharge, upwards of two months. During this time another opening burst about an inch below the former, which discharged a quantity of purulent matter, and a number of exfoliations came away of the irregular appearance as those aforementioned.

Having continued the bark for some time, she gradually recovered her strength; and from that time till the date of this paper, she has not experienced the smallest degree of pain or inconvenience from her breast, or rib which had been fractured.

This patient was also affected with a large steatomatous tumour on the right side of her neck, which had been increasing near fifteen years; it was much larger at its basis than any other part, and extended from the right mastoid process to the clavicle on the same side; and from all the vertebræ of the neck to the mastoid muscle, under which a part of the tumour was situated. It was not attended with pain, but had an unpleasant appearance. Various external remedies had been applied, but effected no sensible alteration or diminution of its size.

During her illness she rested much on that part of the tumour which was most prominent: this circumstance probably accelerated the formation of pus. On observing this tendency, every means were used to promote the suppurative process by emollient cataplasms applied warm, and renewed two or three times in twenty-four hours, in order to convert the substance forming the steatoma into a purulent abscess.

About ten days after the commencement of this operation, there appeared a fluctuation of pus in some parts of the tumour, which I requested to open with a lancet. The patient refused to submit to this operation, but had no objection to the caustic, which was immediately applied to the most depending part of the abscess. And, at this time, and several months after, large lumps of a suety substance resembling tallow, by means of gentle pressure, was discharged from the orifice, with now and then a quantity of purulent matter tinged with blood. During the inflammatory state the application of an emollient poultice was continued.

It has been generally observed, with respect to steatomatous tumours, that it is absolutely necessary that the whole should be removed if possible in the operation, and not not to trust to the effects of dressings: for, if any part is left behind, there have been instances of the tumour arriving at its former size in a few months, and where a repetition of the operation became necessary.

However, in this case, it was impossible to remove the whole at once, as there remained a considerable degree of inflammation. But by means of gentle pressure, during the time the discharge continued, the substance was effectually removed. Since that time, there has been no appearance of any enlargement, and scarcely a scar now remains perceptible.

Scrophulous induration and enlargement are easily distinguished from a cancerous affection of the part, by not being attended with pain, even when greatly increased in size. If it tends to suppuration, it has not in general that craggy feel and unevennes of surface peculiar to cancer ready to burst; and when it suppurates, discharges good pus, which cancer never does. An induration from external violence, affecting the breast, requires the most careful attention. But we are not possessed of any means of knowing that an induration so occasioned will terminate in cancer, if it does not yield to the proper treatment in order to disperse it.

When such induration or scirrhus is occasioned by external injury, it is rather a favourable circumstance, and a presumption that it will not terminate in cancer; and when such induration, or scirrhus, immediately succeeds the external injury, it is a more favourable appearance than when it attacks the part some considerable time after.

In either case, it may remain without pain or unfavourable symptoms for several years, and may quickly become dangerous and alarming, attended with the characteristic symptoms of cancer, and that the external injury was the exciting cause, without the occurrence of which, the person might have passed through life without any inconvenience from the disease; it is, therefore, necessary to be guarded in our prognosis concerning an induration thus occasioned.

*Bath, September*, 1803.

